# Statin activation of skeletal ryanodine receptors (RyR1) is a class effect but separable from HMG‐CoA reductase inhibition

**DOI:** 10.1111/bph.15893

**Published:** 2022-08-02

**Authors:** Chris Lindsay, Maria Musgaard, Angela J. Russell, Rebecca Sitsapesan

**Affiliations:** ^1^ Department of Pharmacology University of Oxford Oxford UK; ^2^ Structural Bioinformatics and Computational Biochemistry, Department of Biochemistry University of Oxford Oxford UK; ^3^ OMass Therapeutics Oxford UK; ^4^ Department of Chemistry, Chemistry Research Laboratory University of Oxford Oxford UK

**Keywords:** Ca^2+^‐release, myopathy, ryanodine receptor, RyR1, single‐channel, statin

## Abstract

**Background and Purpose:**

Statins, inhibitors of HMG‐CoA reductase, are mainstay treatment for hypercholesterolaemia. However, muscle pain and weakness prevent many patients from benefiting from their cardioprotective effects. We previously demonstrated that simvastatin activates skeletal ryanodine receptors (RyR1), an effect that could be important in initiating myopathy. Using a range of structurally diverse statin analogues, we examined structural features associated with RyR1 activation, aiming to identify statins lacking this property.

**Experimental Approach:**

Compounds were screened for RyR1 activity utilising [^3^H]ryanodine binding. Mechanistic insight into RyR1 activity was studied by incorporating RyR1 channels from sheep, mouse or rabbit skeletal muscle into bilayers.

**Key Results:**

All UK‐prescribed statins activated RyR1 at nanomolar concentrations. Cerivastatin, withdrawn from the market due to life‐threatening muscle‐related side effects, was more effective than currently‐prescribed statins and possessed the unique ability to open RyR1 channels independently of cytosolic Ca^2+^. We synthesised the one essential structural moiety that all statins must possess for HMG‐CoA reductase inhibition, the *R*‐3,5‐dihydroxypentanoic acid unit, and it did not activate RyR1. We also identified five analogues retaining potent HMG‐CoA reductase inhibition that inhibited RyR1 and four that lacked the ability to modulate RyR1.

**Conclusion and Implications:**

That cerivastatin activates RyR1 most strongly supports the hypothesis that RyR1 activation is implicated in statin‐induced myopathy. Demonstrating that statin regulation of RyR1 and HMG‐CoA reductase are separable effects will allow the role of RyR1 in statin‐induced myopathy to be further elucidated by the tool compounds we have identified, allowing development of effective cardioprotective statins with improved patient tolerance.

**Abbreviations**
CCD: central core disease
HMG‐CoA: 3‐hydroxy‐3‐methylglutaryl CoA
LDL: low density lipoprotein
MH: malignant hyperthermia

**What is already known**

- Simvastatin activates single RyR1 channels and increases sarcoplasmic reticulum (SR) Ca^2+^‐release in muscle cells.
- Cerivastatin induces Ca^2+^‐release from the SR in mouse and rat skeletal muscle fibres.

**What does this study add**

- RyR1 activation is a class effect of all commonly‐prescribed statins. Cerivastatin is particularly effective.
- Potent HMG‐CoA inhibition is achievable without also activating RyR1.

**What is the clinical significance**

- Cerivastatin, associated with the most severe side effects, is the strongest activator of RyR1.
- Statins that do not activate RyR1 may allow many more patients to benefit.

## 1 INTRODUCTION

Statins are among the most widely prescribed drugs worldwide, as treatment for both the primary and secondary prevention of cardiovascular events (Nishimura et al., 2014; Oates et al., 1988). Global sales are soon predicted to reach $1 trillion, while atorvastatin was the most widely prescribed drug in the United Kingdom in 2018, with over 37% of the adult population eligible for treatment (Ioannidis, 2014; Ueda et al., 2017). Statins are prescribed to reduce blood cholesterol levels and the mechanism of action of all statins relies on competitive inhibition of the enzyme catalysing the rate‐determining step of cholesterol synthesis, HMG‐CoA reductase. The decrease in cholesterol production also promotes the redirection of cholesterol to the liver via low density lipoprotein cholesterol (LDL) receptors, thus further lowering plasma cholesterol levels (Brown & Goldstein, 2004). Large‐scale clinical trials have demonstrated that statins can promote up to 60% reductions in plasma LDL levels and have shown that their use is associated with a significant decrease in the risk of cardiovascular events (Collins et al., 2016; Vaughan & Gotto, 2004).

**FIGURE 1:**
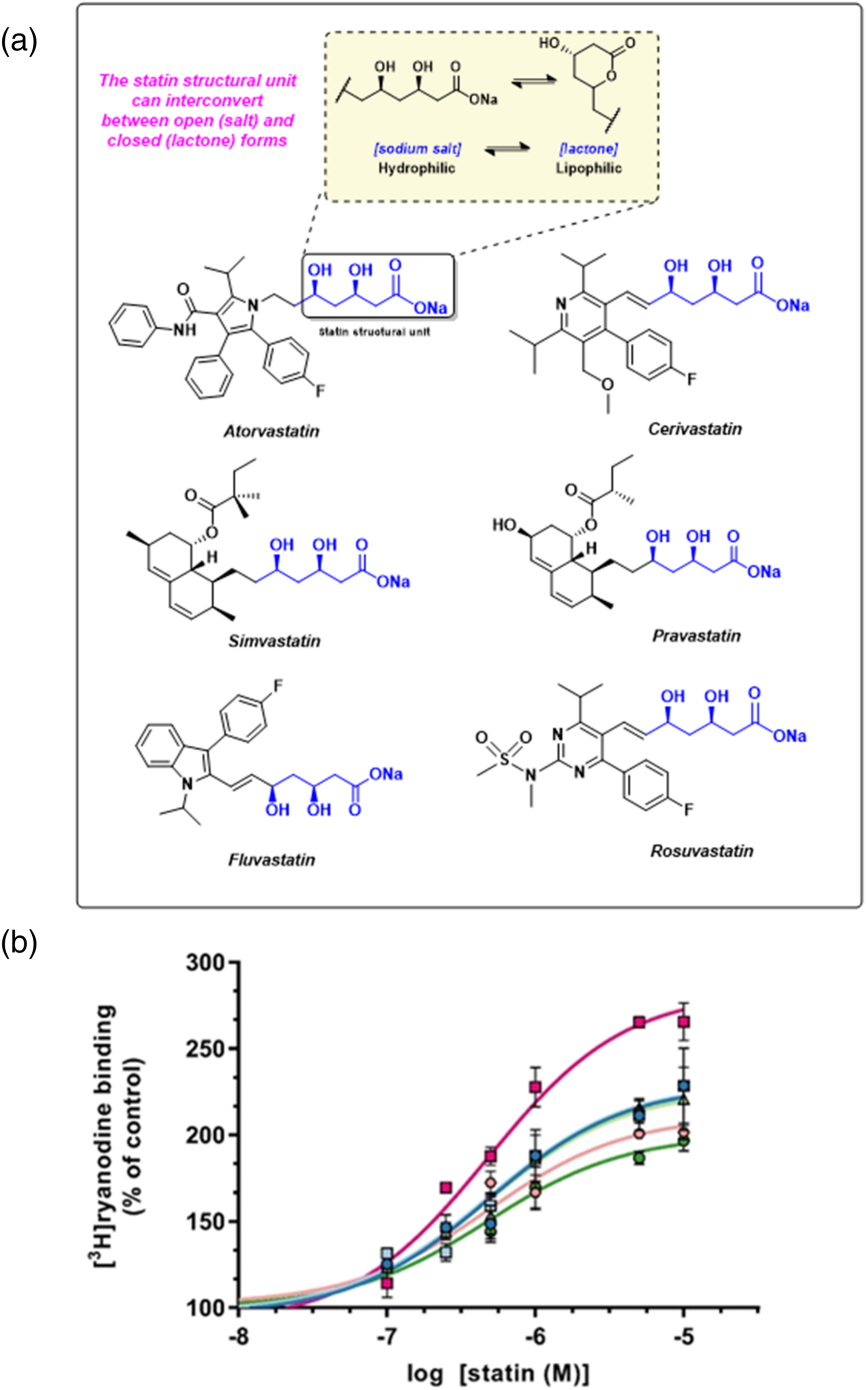
The effects of commonly prescribed statins on ryanodine RyR1 channel activity. (a) The chemical structures of atorvastatin, cervistatin, simvastatin, pravastatin, fluvastatin and rosuvastatin are shown. In each structure, the essential statin structural unit required for activity at HMG‐CoA reductase is indicated in blue. This unit can exist in both an open and ring‐closed form as illustrated in the inset. (b) Increase of [^3^H]ryanodine binding to sheep skeletal muscle SR membrane vesicles by commonly prescribed statins indicated as a percentage of control binding levels (10 μM Ca^2+^ as sole activator). Atorvastatin (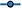; EC_50_ = 0.76 μM, E_max_ = 229%) cerivastatin (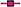; EC_50_ = 0.42 μM, E_max_ = 283%), simvastatin (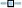; EC_50_ = 0.70 μM, E_max_ = 230%), pravastatin (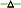; EC_50_ = 0.44 μM, _max_ = 227%), fluvastatin (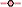; EC_50_ = 0.42, E_max_ = 211%) and rosuvastatin (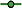; EC_50_ = 0.49 μM, E_max_ = 200%) are shown. Mean ± SEM; *n* = 5; where error bars are not visible, they are within the symbol

There are currently five statins prescribed in the United Kingdom: atorvastatin (Lipitor), simvastatin (Zocor), pravastatin (Lipostat), fluvastatin (Lescol) and rosuvastatin (Crestor). They can be broadly grouped into two categories based on their chemical structures: those based upon a natural product decalin core (e.g., simvastatin) and those that are based upon a fully synthetic polycyclic structure (e.g., atorvastatin) (Istvan, 2003). However, while structurally distinct, all statins share the same basic interactions with HMG‐CoA reductase (Schachter, 2005). There is one minimum essential molecular moiety, a required structural element, that all statins must possess if they are to bind to and inhibit HMG‐CoA reductase. This is the (*R*)‐3,5‐dihydroxypentanoic acid unit, present also in HMG‐CoA, the natural substrate of HMG‐CoA reductase (Istvan & Deisenhofer, 2001). This required structural element can exist in both a lactone (prodrug) form and an open ring (acid) form (Figure 1a, inset); however, the equilibrium heavily favours the open form at physiological pH (Taha et al., 2016). The lactone form is inactive as an inhibitor of HMG‐CoA reductase, but we have previously shown that both forms of simvastatin can activate RyR1 (Venturi et al., 2018). Indeed, the lactone form of simvastatin has been found to be more rapidly taken up by skeletal muscle cells, leading to increased cytotoxicity compared to the open salt/acid form (Taha et al., 2016).

Despite their proven efficacy in lowering plasma cholesterol levels, not all patients can tolerate the dose of statins required to achieve a therapeutic benefit (Ward et al., 2019). This can be due to skeletal muscle related side effects that are an established feature of statin treatment. These include myalgia/muscle pain, myopathy linked to a rise in creatine kinase, and, in very severe cases, rhabdomyolysis, which can result in death (Tomaszewski et al., 2011). The exact prevalence of statin side effects is a matter of debate, as controlled placebo‐based trials have reported incidence levels for myopathy of between 1% and 10%, while observational studies report values as high as 30% (Adhyaru & Jacobson, 2018; Bruckert et al., 2005; Parker et al., 2013). There is also significant evidence that the co‐administration of drugs such as gemfibrozil can increase plasma statin concentration and increase the risk of adverse effects (Jacobson & Zimmerman, 2006). The most serious cases of rhabdomyolysis appear to occur in approximately 1 in 10,000 patients on statin treatment (Newman & Tobert, 2018). Most adverse effects occurred with cerivastatin (Baycol), a statin withdrawn from the market in 2001, as postmarket surveillance reported 52 deaths had occurred from rhabdomyolysis as a result of its use (Furberg & Pitt, 2001). While it is fortunate that the majority of reported side effects are mild, there is significant evidence that they result in a decrease in patient adherence to medication, reducing the effectiveness of statins in the clinical setting (Mann et al., 2010).

The core driver of statin side effects has been the subject of intense research, with a number of proposed mechanisms. These include disruption of mitochondrial respiration (Dohlmann et al., 2019), depletion of coenzyme Q_10_ levels (Deichmann et al., 2010), reduction of cellular structural integrity (Draeger et al., 2006) and reduced levels of downstream metabolites such as farnesyl pyrophosphate (Abd & Jacobson, 2011). However, although muscle‐related side‐effects are associated with all statins, their severity or incidence does not appear to correlate with relative ability to inhibit HMG‐CoA reductase, suggesting that they must be caused by a mechanism independent from cholesterol depletion (Armitage, 2007). It instead appears that the disruption of Ca ^2+^ signalling in skeletal muscle cells may be important. It is well established that muscle contraction relies on a coordinated cycling of Ca^2+^ release and re‐uptake from/to the sarcoplasmic reticulum (SR) (Gordon et al., 2000; Rios & Brum, 1987). Statins have been found to interrupt this cycle by provoking inappropriate Ca^2+^‐release from the SR. Acute treatment triggers marked Ca^2+^‐release from the SR both in individual rat SR vesicles (Inoue et al., 2003) and in whole muscle fibres (Sirvent et al., 2005), while chronic statin treatment in rats appears to increase the resting Ca^2+^ concentration ([Ca^2+^]_i_) in muscle cells (Liantonio et al., 2007). It had been suggested that statins could operate via the skeletal muscle ryanodine receptor (RyR1), the main SR Ca^2+^‐release channel in skeletal muscle), and we recently reported that this is indeed the case ((Sirvent et al., 2005; Venturi et al., 2018). We showed that simvastatin directly activates single skeletal muscle RyR1 channels incorporated into artificial membranes under voltage clamp conditions (Venturi et al., 2018). Simvastatin also stimulated the binding of [^3^H]ryanodine to RyR1, demonstrating that populations of RyR1 channels in their native membranes are also activated by this statin (Venturi et al., 2018).

Given the association between RyR1 dysfunction, myopathy, and statin‐induced Ca^2+^‐release, we hypothesised that statin‐induced RyR1 activation is a contributing factor to the muscle‐related side effects of patients prescribed statin treatment (Lindsay, 2018; Venturi et al., 2018). We now report that RyR1 activation is a class effect, common to all statin drugs currently approved for use in the United Kingdom. We further describe the identification of statin molecules, structural analogues of atorvastatin, which are devoid of RyR1 activation, as tool compounds to inform the development of the next generation of statin drugs.

## 2 METHODS

### 2.1 Animals and ethical approval

Single‐channel experiments were performed using either mouse, rabbit or sheep skeletal muscle tissue as indicated in individual figure legends. All [^3^H]ryanodine binding experiments were performed using tissue derived from sheep. We selected sheep tissue for this work as it could be obtained from a commercial abattoir, therefore reducing unnecessary use of animals without compromising the integrity of the data. Sheep tissue was obtained from a commercial abattoir that complies with the requirements of the Department for Environment, Food & Rural Affairs. Sheep were Suffolk breed, aged between 8 and 10 years, and of mixed sex. Sheep were sacrificed by captive bolt, which was performed by a registered veterinarian. Male New Zealand white rabbits (1–6 kg, aged 1–3 years, mixed sex) were obtained from a commercial supplier (Charles Rivers, Alderly Park, UK). Rabbits were housed in individual cages with controlled temperature, lighting, and humidity. Food and water were provided ad libitum. Mice (C57BL/6, mixed sex, aged 3–6 months) were obtained from a commercial supplier (Charles Rivers, Alderly Park, UK). Mice were housed in groups of up to six in a specific pathogen‐free unit in individually ventilated cages on a 12‐h light/dark cycle with free access to food and water. All work was performed in accordance with the Directive 2010/63/EU of the European Parliament with local approval of the Animal Research Committee according to the regulations on animal experimentation at the University of Oxford. Animal studies are reported in compliance with the ARRIVE guidelines (Percie du Sert et al., 2020) and with the recommendations made by the *British Journal of Pharmacology* (Lilley et al., 2020).

### 2.2 Preparation of SR vesicles

Heavy sarcoplasmic reticulum (HSR) vesicles were prepared from skeletal (RyR1) muscle tissue as previously reported (Sitsapesan & Williams, 1990).

### 2.3 Single‐channel recordings

Skeletal HSR vesicles were incorporated into planar phospholipid bilayers as previously described (Sitsapesan et al., 1991). Voltage‐clamp conditions were used to measure the current through the RyR channels, with the *trans* (luminal) chamber voltage‐clamped at ground. The recording solutions used were 250‐mM HEPES, 80‐mM Tris, 10‐μM free Ca^2+^ and pH 7.2 on the cytosolic side and 250‐mM glutamic acid, 10‐mM HEPES and ≈50‐mM free Ca^2+^ on the luminal side, both at 21°C. The statin compound under investigation was added to the cytosolic or luminal chamber as required. The free [Ca^2+^] was maintained by the addition of EGTA and CaCl_2_ solution and measured using a Orion 93‐20 Ca^2+^ electrode (Thermo Fisher Scientific, Horsham and Loughborough, UK) as previously described (Sitsapesan et al., 1991). The pH of solutions was determined using a Ross‐type pH electrode (Orion 81‐55, Thermo Fisher Scientific, UK) as previously described (Sitsapesan et al., 1991). To lower free cytosolic [Ca^2+^] below subactivating levels, 1‐mM EGTA was added. This reduced free [Ca^2+^] below measurable levels, and the concentration was calculated using the MaxChelator programme (https://somapp.ucdmc.ucdavis.edu/pharmacology/bers/maxchelator/index.html) (Chris Patton, Stanford, USA) to <1 nM.

### 2.4 Single‐channel analysis

Single‐channel analysis was performed as described previously (Sitsapesan et al., 1991; Sitsapesan & Williams, 1990). Briefly, recordings were low‐pass filtered at 800 Hz and digitised at 20 kHz. Open probability (Po) was determined over 3 min of continuous recording, and this is the value reported in all figures. Where several channels were present in a recording, the average Po based on *N* channels is reported according to the equation:

$$\text{average}\;P_{O}=\frac{NP_{O}}{N}=\frac{P_{O1}+P_{O2}+P_{O3}+\ldots nP_{O_{n}}}{N}$$

where $P_{O_{1}}$, $P_{O_{2}}$ and $P_{O_{3}}$ are the probability of dwelling in the first, second or third channel levels, respectively. Lifetime analysis was performed when only one channel was present in the bilayer using Clampfit 10.6 (Molecular Devices, USA).

Lifetime distributions were constructed using Clampfit 10.6 (Molecular Devices, USA) and fitted to a probability density function (pdf) by the method of maximum likelihood (Colquhoun & Sigworth, 1995) according to the equation:

$$f\left(t\right)=\sum_{i}^{N}a_{i}f_{0}\left(t-\ln\;\tau_{i}\right)$$

where *i* is the number of exponential components of the distribution, *τ*_*i*_ are the time constants and *a*_*i*_ are the fractions of the total events represented by the *i*th component. The set of parameters was adjusted by the maximum likelihood iterative algorithm until the optimum value of the log of likelihood L was reached (Blatz et al., 1986). To count the number of ion channels in the bilayer, ≥1‐mM ATP was added at the end of the experiment to increase Po to high levels.

### 2.5 [^3^H]ryanodine binding

HSR vesicles (100–130 μg/μl^−1^) were incubated at 37°C in 250‐mM HEPES, 80‐mM Tris, 10‐μM free Ca^2+^, pH 7.2, and 5‐nM [^3^H]ryanodine for 90 min. Samples were filtered through glass 25‐mm‐diameter microfibre filters (VMR, UK), and the filters were washed with 10 ml of ice‐cold water. The filters were dissolved in 20 ml of scintillation fluid (EmulsiferSafe, Perkin Elmer, UK) and counted. Nonspecific binding was determined from a sample with an additional 5‐μM unlabelled ryanodine added. All experiments were performed in triplicate. To control for variation in RyR levels between SR preparations, 5‐nM [^3^H]ryanodine binding data (Emax: maximum number of binding sites in presence of statin) were normalised relative to control binding levels (250‐mM HEPES, 80‐mM Tris, 10‐μM free Ca^2+^ and pH 7.2), and expressed as a percentage.

### 2.6 Data and statistical analysis

The data and statistical analysis in this study comply with the recommendations on experimental design and analysis in pharmacology (Curtis et al., 2018). Data analysis was performed using Graphpad Prism 7 (San Diego, California, USA). Data were assessed for normality using the D'agostino and Pearson test. Data are expressed as mean ± SEM, and where *n* ≥ 5, comparisons are made using a one‐way ANOVA using a mixed linear model with random effects, followed by Dunnett's post hoc test. A *p* value of <0.05 was taken as significant throughout. Variations in *n* numbers reported for single‐channel experiments are due to bilayers breaking during the experiment, precluding further measurements being taken. Post hoc analyses were only performed if *F* was significant, and there was no significant variance in homogeneity. In all cases, data were obtained from at least five different skeletal membrane preparations (using tissue derived from one animal each for sheep and five animals for mouse). Single‐channel experiments were blinded to compound identity. No outlier analysis was performed, and no data was excluded from analysis. Formal power calculations were not undertaken; however, group sizes were determined by taking into account experience of our previous investigations with statins and to avoid an unnecessary number of animals in compliance with the principles of the ‘three Rs’. For all experiments, SR preparations were derived from at least five animals. Animals of the same species were selected at random for SR preparations, and experiments were performed after random incorporation of RyR1 channels into bilayers or SR vesicles into test tubes for [^3^H]ryanodine binding experiments.

### 2.7 Materials

Statin sodium salts were obtained from CarboSynth Ltd (Compton, UK). [9, 21(*n*)‐^3^H]ryanodine was obtained from Amersham Biosciences (Buckinghamshire, UK). All other chemicals were purchased from Sigma‐Aldrich (Dorset, UK) or VWR (Poole, UK). The atorvastatin analogues in Figure 7 were kindly provided by Pfizer Inc. via their compound transfer program. All statin compounds were provided as solids. The statin analogues from *Pfizer* were supplied as solids with defined >95% purity by LCMS (Pfizer). Water was deionised (Millipore, Harrow, UK) and all single channel recording solutions were filtered through a membrane of 0.45 μM diameter before use. All statin compounds were reconstituted in water to a concentration of 10 mM immediately prior to use.

### 2.8 Nomenclature of targets and ligands

Key protein targets and ligands in this article are hyperlinked to corresponding entries in the IUPHAR/BPS Guide to PHARMACOLOGY http://www.guidetopharmacology.org and are permanently archived in the Concise Guide to PHARMACOLOGY 2021/22 (Alexander, Fabbro et al., 2021; Alexander, Kelly et al., 2021; Alexander, Mathie et al., 2021).

## 3 RESULTS

### 3.1 All commonly prescribed statins activate RyR1

Ryanodine binds with high affinity to RyR channels only when the channels are open (Lai & Meissner, 1989). The probability of ryanodine binding to an RyR channel therefore increases as Po increases and hence [^3^H]ryanodine binding has become a standard assay to estimate the Po of populations of RyR channels in their native membranes (Chu et al., 1990). The statins that are currently prescribed in the United Kingdom (Figure 1a), as well as cerivastatin, were therefore investigated for their ability to increase [^3^H]ryanodine binding to skeletal HSR vesicles containing RyR1 (Figure 1a). It was found that all of these statins increased [^3^H]ryanodine binding at similar nanomolar concentrations, indicating that they may bind to RyR1 with similar affinity (Figure 1b). The stimulation of binding was significant by 100 nM with all statins except rosuvastatin and pravastatin and, with these, binding was significantly elevated by 250 nM. Cerivastatin, which was removed from the market due to adverse effects (Maggini et al., 2004) was more effective than the other statins, causing the highest maximum level of [^3^H]ryanodine binding (Figure 1b).

### 3.2 Atorvastatin and cerivastatin activate single RyR1 channels

**FIGURE 2:**
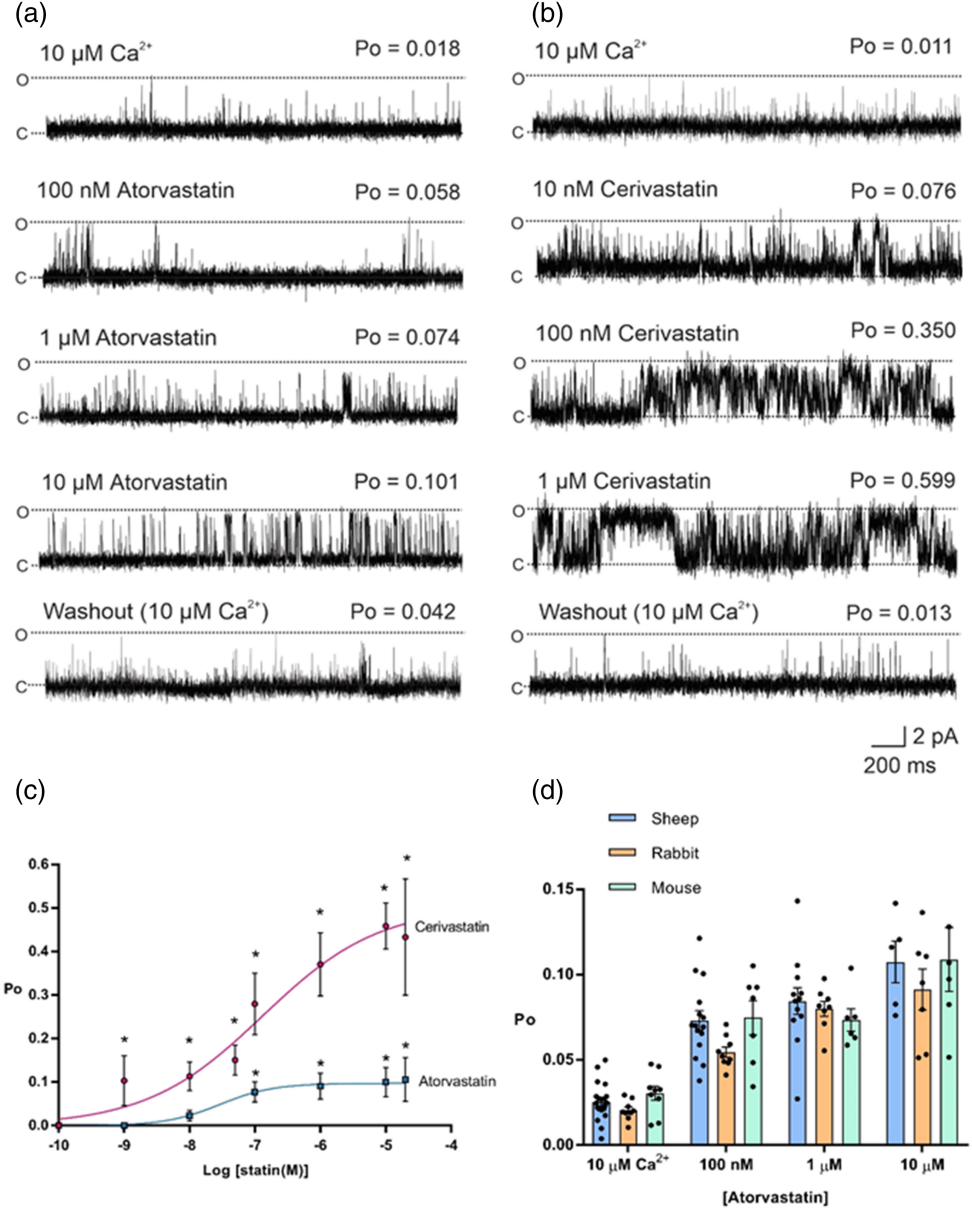
The effects of atorvastatin and cerivastatin on ryanodine RyR1 single channel function. Representative sheep skeletal RyR1 single channel fluctuations in the presence of 10‐μM cytosolic Ca^2+^ alone (top trace) and after addition of cytosolic atorvastatin (a) or cerivastatin (b) as indicated (middle traces) and after washout of the cytosolic chamber to control conditions (bottom trace). O and C indicate the open and closed channel levels, respectively. The Po above each trace refers to the value determined over 3 min. (c) Concentration–response relationships for the activation of RyR1 channels by cytosolic atorvastatin (EC_50_ = 29 nM, E_max_ = 0.10) and cerivastatin (EC_50_ = 130 nM, E_max_ = 0.50). Mean ± SEM; *p < .05 relative to control, *n* (control: 22, atorvastatin: 10 nM 21; 50 nM 19; 100 nM 18; 1 μM 10; 10 μM 8; 20 μM 8, cerivastatin 10 nM 20; 50 nM 20; 100 nM 18; 1 μM 15; 10 μM 14; 20 μM 9). (d) Mean Po values for RyR1 channels in the presence 10‐μM Ca^2+^ and after addition of atorvastatin (concentrations indicated) for RyR1 channels derived from sheep (blue), rabbit (orange) and mouse (green) skeletal muscle, respectively. Mean ± SEM; *n* = sheep: control 22; 100 nM 18; 1 μM 10; 10 μM 8; mouse *n* = 6 throughout; rabbit *n* = 9 throughout

[^3^H]ryanodine binding can provide an estimate of the activity of a population of RyR channels, but single‐channel analysis is required to investigate the mechanism of action. We therefore incorporated sheep heavy SR vesicles containing RyR1 channels into planar lipid bilayers under voltage‐clamp conditions. Atorvastatin and cerivastatin were selected for single‐channel experiments, as atorvastatin is the most widely prescribed among the available drugs, while cerivastatin caused fatalities in the patient population (Furberg & Pitt, 2001) and, in the above [^3^H]ryanodine binding assay, was the strongest activator of RyR1. In the presence of 10‐μM free cytosolic Ca^2+^, both statins caused a concentration‐dependent increase in Po when added to the cytosolic chamber as shown in the representative experiments (Figure 2a,b). The comparative single‐channel traces demonstrate that cerivastatin is more effective at each concentration tested, and this is confirmed in the concentration–response relationships shown in Figure 2c. It is clear that both statins are partial agonists, but that cerivastatin has a greater ability (efficacy) to increase Po to high levels. As the example traces show, activation by both statins was reversible when control conditions were restored by perfusion, although a longer perfusion time was required to remove cerivastatin (3 min vs. 2 min for atorvastatin). This may suggest that cerivastatin dissociates slower from RyR1 or reflect its greater tendency to partition into membranes (Galiullina et al., 2019). After perfusion, Po was 0.0138 ± 0.0072, *n* = 6, and 0.0138 ± 0.0073, *n* = 5 for atorvastatin and cerivastatin, respectively.

**FIGURE 3:**
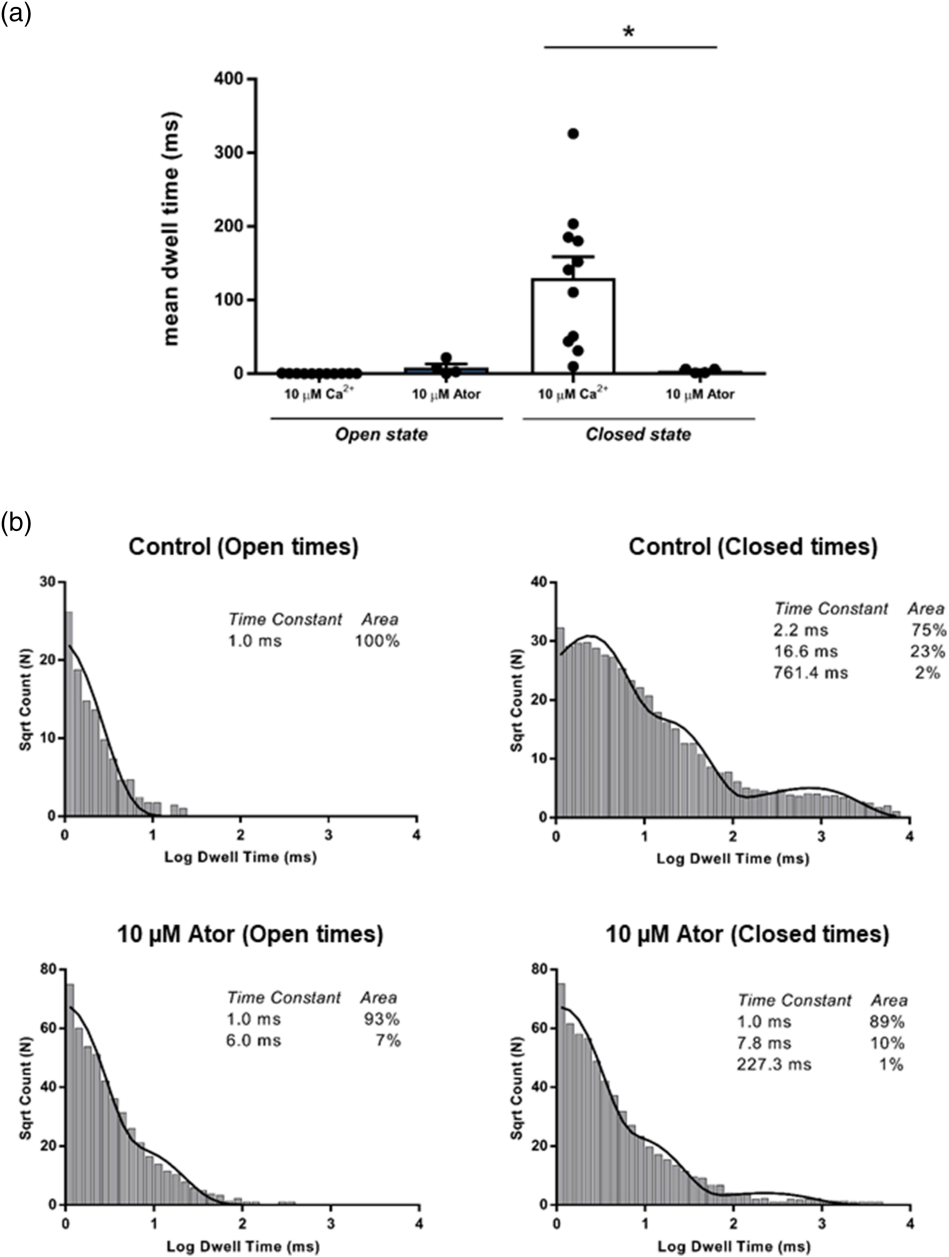
Lifetime analysis of statin induced ryanodine RyR1 activity. (a) The effect of cytosolic atorvastatin on RyR1 mean open and closed times. Mean ± SEM; *n* = 5, *p < .05. (b) Open and closed life‐time distributions and probability density functions for a representative RyR1 channel in control conditions (10‐μM Ca^2+^, top) and after addition of 10‐μM atorvastatin (Ator)(bottom). The time constants (τ) and percentage areas are shown

To investigate the mechanism underlying the atorvastatin increase in Po, we performed lifetime analysis when only a single channel was present in the bilayer. Figure 3a shows that the mean closed time was significantly decreased after addition of 10‐μM atorvastatin whereas no significant alteration to mean open time was observed. Further detail in these lifetime changes can be seen in the representative example of open and closed lifetime distributions in the presence of 10‐μM cytosolic Ca^2+^ (control) and after addition of 10‐μM atorvastatin (Figure 3b). While there is an additional second longer time component in the pdf for open events, it can be seen that the major changes take place in the duration of the closed events. In the presence of atorvastatin, all three time constants are reduced and a greater proportion of all the events occur to the shortest time constant demonstrating that the primary mechanism by which atorvastatin increases Po is by increasing the frequency of openings even though there is a trend towards a slight increase in open times. This is similar to our findings with simvastatin (Venturi et al., 2018)

Addition of atorvastatin to the luminal chamber did not cause a significant increase in Po, even at high concentrations, (control Po: 0.0295 ± 0.0075, *n* = 5; 100‐μM luminal atorvastatin: 0.0318 ± 0.0128, *n* = 5). A similar observation was found with simvastatin (Venturi et al., 2018).

We also investigated whether there were any marked species differences in the ability of statins to activate RyR1. Atorvastatin increased the Po of single RyR1 channels derived from sheep, mouse and rabbit skeletal muscle to similar levels (Figure 2d). This is consistent with the high level of similarity in the RyR1 gene between these species (Hakamata et al., 1992). The sequence identity of each of these species with respect to human RyR1 (P21817) is sheep 91% (W5P6U4), rabbit 96% (P11716) and mouse 95% (E9PZQ0).

**FIGURE 4:**
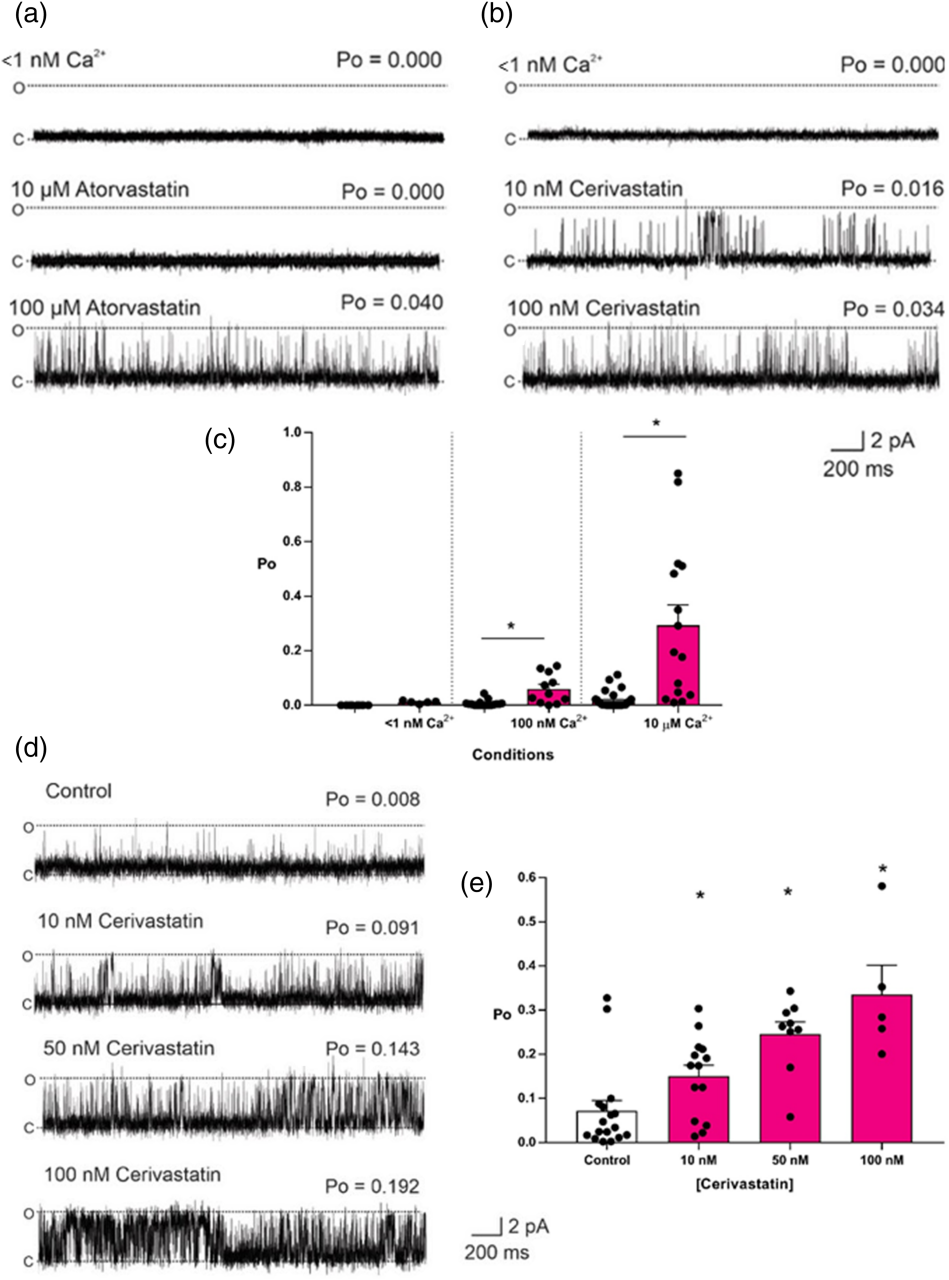
The Ca^2+^‐dependency of statin ryanodine RyR1 activity. Representative sheep skeletal muscle RyR1 single channel fluctuations at subactivating [Ca^2+^] < 1 nM Ca^2+^ (top trace) are shown and after addition of cytosolic atorvastatin (a) or cerivastatin (b) as indicated. Note the difference in concentrations between atorvastatin and cerivastatin. O and C indicate the open and closed channel levels, respectively. The Po above each trace refers to the value determined over 3 min. (c) Mean Po values for RyR1 channels before (white bars) and after (pink bars) addition of 100 nM cerivastatin at the free [Ca^2+^] indicated. Mean ± SEM; *p < .05 relative to respective controls; *n* (<1 nM Ca^2+^ 11; 100 nM 9; 10 μM 6). (d) Representative sheep skeletal muscle RyR1 single channel fluctuations under control conditions (100‐nM cytosolic Ca^2+^, 1‐mM free Mg^2+^ and 5‐mM ATP) (top trace) and after addition of cytosolic cerivastatin as indicated. O and C indicate the open and closed channel levels, respectively. The Po above each trace refers to the value determined over 3 min. (e) Mean RyR1 Po values under control conditions (100‐nM cytosolic Ca^2+^, 1‐mM free Mg^2+^ and 5‐mM ATP) and after the subsequent addition of cerivastatin as indicated. Mean ± SEM; *p < .05; *n* (control 32; 10 nM 28; 50 nM 25; 100 nM 15)

We have previously demonstrated that activation of RyR channels by simvastatin is highly Ca^2+^‐dependent (Venturi et al., 2018). We therefore investigated if activation of RyR1 by atorvastatin and cerivastatin was also dependent on cytosolic Ca^2+^. Figure 4a,b shows typical experiments where the cytosolic free [Ca^2+^] was lowered to a subactivating level (<1 nM) to abolish channel openings (top traces). Under these conditions, the figure shows that subsequent additions of low nanomolar concentrations (10 nM) of cerivastatin can still induce channel openings whereas high micromolar levels of atorvastatin (similar to simvastatin: Venturi et al., 2018) are required to produce any activation. Thus, cerivastatin has the ability to open RyR1 in a Ca^2+^‐independent manner at nanomolar concentrations, a property that simvastatin and atorvastatin do not possess. Nanomolar concentrations of simvastatin and atorvastatin absolutely require the presence of cytosolic Ca^2+^ before they can stimulate the opening of RyR1. The ability of low nanomolar concentrations of cerivastatin to activate RyR1 in this Ca^2+^‐independent manner is of pathological significance as this will increase the likelihood of excess Ca^2+^ leak from skeletal muscle SR at rest and during contraction. To understand the relative potentiation of RyR1 channel openings that cerivastatin causes at subactivating [Ca^2+^] (<1 nM), at the resting free [Ca^2+^] in a cell (100 nM), and during a Ca^2+^‐release event (10 μM), we compared the effects of a low concentration of cerivastatin (100 nM) at these levels of free [Ca^2+^] as shown in Figure 3c. It can be seen that at the cellular resting free [Ca^2+^] of 100 nM, cerivastatin is capable of promoting significant channel activation but that the magnitude of activation becomes greater with increasing levels of cytosolic Ca^2+^. This potent effect of cerivastatin could be responsible for the disruption to muscle Ca^2+^ homeostasis that has been reported in patients (Rodriguez et al., 2000). If this is the case, then it is important to ascertain if cerivastatin still induces RyR1 openings at resting free [Ca^2+^] of 100 nM in the presence of the physiological regulators, ATP and Mg^2+^. Figure 3d shows a representative experiment performed in the presence of 100‐nM free cytosolic Ca^2+^, 1‐mM free cytosolic Mg^2+^ and 5‐mM ATP. The top trace shows channel gating under these control conditions. It can be seen that cerivastatin produced a concentration‐dependent increase in Po. Figure 3e shows the mean data illustrating the potent nature of the activation by cerivastatin.

**FIGURE 5:**
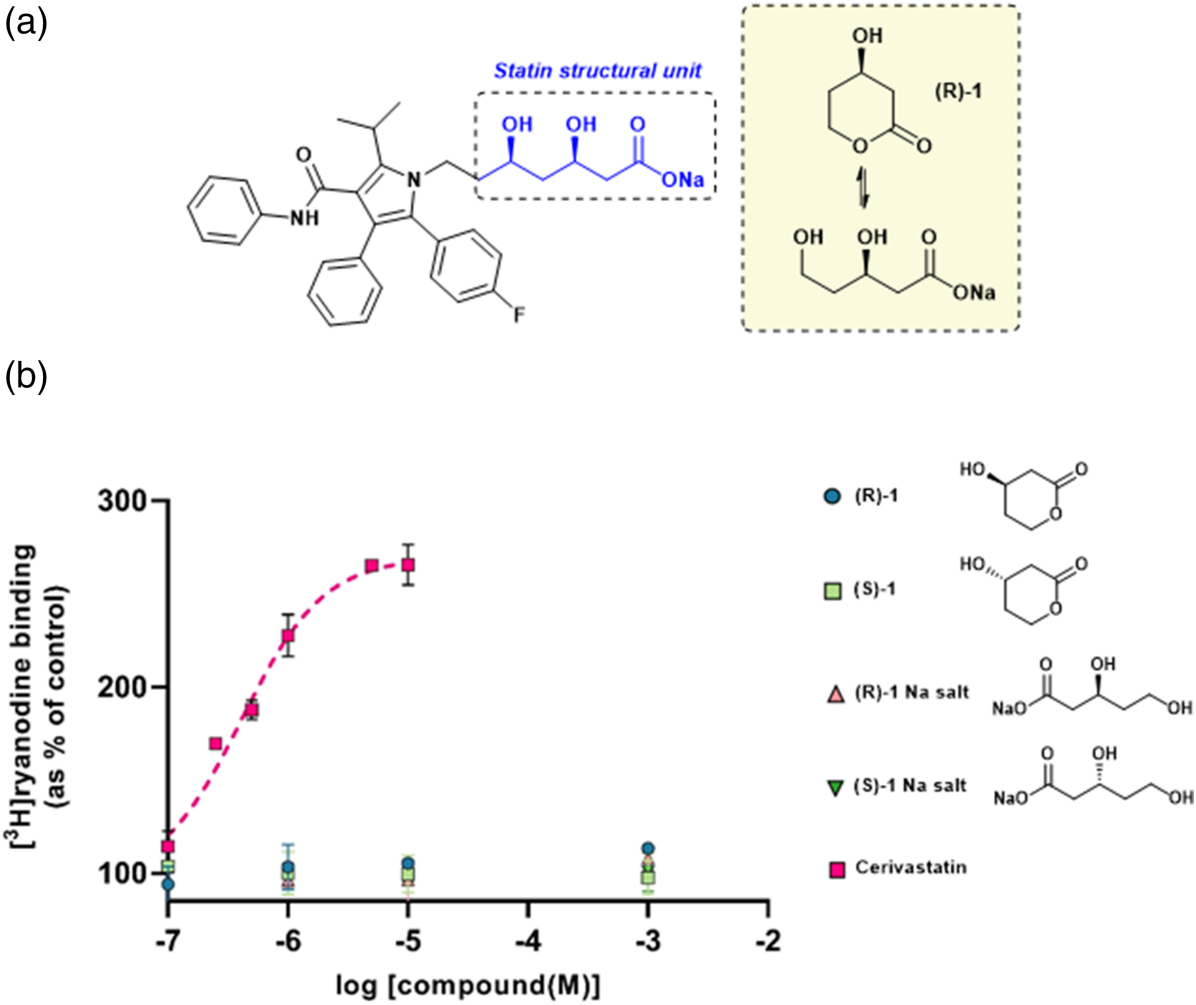
Synthesis of the essential statin structural unit and its effect on ryanodine RyR1 activity. (a) The chemical structure of atorvastatin indicating the essential statin structural unit required for activity at HMG‐CoA reductase (blue) and its comparison to this minimal unit (inset). (b) Increase of [^3^H]ryanodine binding to sheep skeletal SR membrane vesicles by (*R*)‐**1**, (*S*)‐**1**, and their respective sodium salts, indicated as a percentage of control binding levels. Cerivastatin data from Figure 1b is indicated in pink for comparison. Mean ± SEM; *n* = 5; where error bars are not visible, they are within the symbol

As described in Section 1, the minimum essential statin moiety, the (*R*)‐3,5‐dihydroxypentanoic acid unit, which we now term (*R*)‐**1** (Figure 5a) (Istvan & Deisenhofer, 2001), may interconvert between closed lactone and open salt/acid forms. If the goal of producing a HMG‐CoA reductase inhibitor that lacks the ability to activate RyR1 is to be successful, it is important that the minimum essential statin moiety itself does not activate RyR1 because we cannot modify this moiety. This moiety was therefore synthesised and investigated for its ability to alter RyR1 gating. (*R*)‐**1** and its corresponding sodium salt were synthesised according to a modified procedure based upon that previously reported by Loubinoux and colleagues (Loubinoux et al., 1995) (Figure S1).

The aldehyde **4** was prepared by TBDPS protection of propane‐1,3‐diol **2** followed by Swern oxidation in 64% yield over two steps, using previously published procedures (Raghavan & Samanta, 2013). Subsequent Evans aldol reaction with oxazolidinone **5** afforded a mixture of diastereomers **6** and **7** in the ratio of 1.65:1 and overall yield of 61%. The low selectivity of this method when utilising isopropyl‐substituted oxazolidinones has been previously reported (Yamashita et al., 2018); however, the resulting diastereomers could be easily separated by flash column chromatography. Subsequent treatment with HF in pyridine afforded the unprotected diastereomers **8** and **9** in good yield. Finally, treatment with Et_3_N for 48 hours and subsequent co‐evaporation with toluene yielded the target compound (*R*)‐**1** and its enantiomer (*S*)‐**1**. The absolute configuration of each was confirmed by use of a chiral shift reagent and by comparison to literature where the configuration was unambiguously determined (Loubinoux et al., 1995). Access to their corresponding sodium salts was achieved by addition of NaOH to pH 7.4, followed by overnight incubation at 37°C, after which LC–MS indicated full conversion.

Figure 5b demonstrates that neither (*R*)‐**1**, (*S*)‐**1**, nor their sodium salts significantly increased the binding of [^3^H]ryanodine to HSR vesicles at concentrations up to 1 mM, indicating that that the statin structural unit does not, alone, activate RyR1. To emphasise this point, the cerivastatin data from Figure 1b is plotted on the graph to illustrate the degree to which cerivastatin increases [^3^H]ryanodine binding under these conditions.

### 3.3 Discovery of statins devoid of RyR1 activation

**FIGURE 6:**
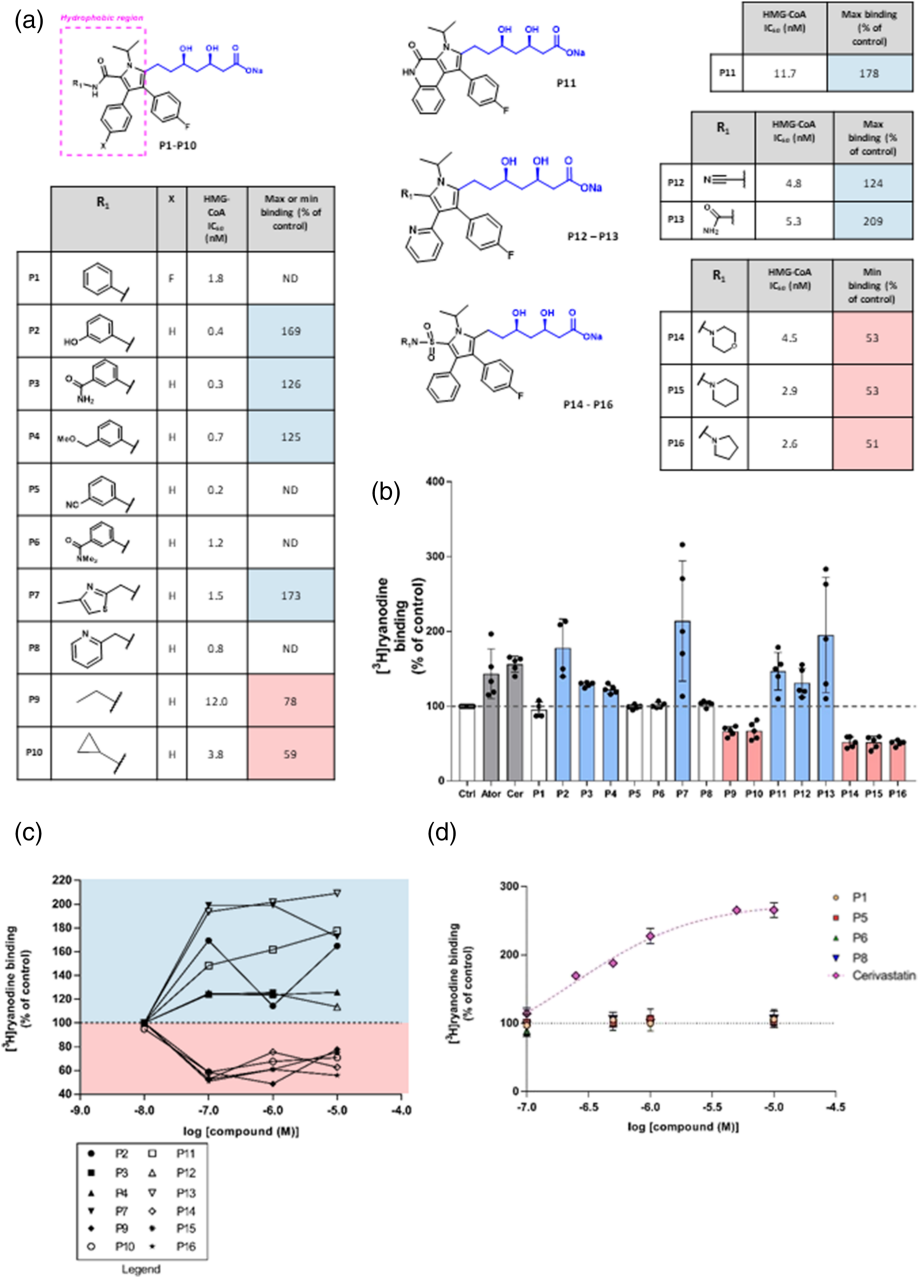
Structure–activity relationship for the activation of ryanodine RyR1 by atorvastatin analogues. (a) Chemical structures of atorvastatin analogues **P1**–**P16**, their experimentally determined IC_50_ values for in vitro HMG‐CoA inhibition (Bratton et al., 2007; Larsen et al., 2007; Park et al., 2008; Pfefferkorn, Choi, et al., 2007; Pfefferkorn, Song, et al., 2007; Suzuki et al., 2001) and the maximum increase (RyR1 activation) or inhibition (RyR1 inhibition) of [^3^H]ryanodine binding to sheep skeletal muscle SR membranes. RyR1 activators are shown in blue, RyR1 inhibitors in pink and those compounds with no effect on RyR1 activity are shown in white. (b) Increase of [^3^H]ryanodine binding for compounds **P1**–**P16** at 100 nM, relative to control binding levels (Ctrl). 100‐nM cerivastatin (Cer) and 100‐nM atorvastatin (Ator) are also shown for comparison. Mean ± SEM; *n* = 5. (c) Line graphs of the relationship between ability to increase [^3^H]ryanodine binding to sheep skeletal muscle SR membranes for activators and inhibitors relative to control binding levels. Compounds with no effect on RyR1 within the given range of concentrations are not shown. The lines have no theoretical significance and are shown for ease of comparison only. Error bars are not shown for clarity. The symbols are shown in the adjacent legend. (d) Concentration–response relationships for P1, P5, P6 and P8 relative to control binding levels are shown. To illustrate the inactivity of these compounds, the cerivastatin data from Figure 1 are also shown (mean ± SEM; *n* = 6)

Following this finding, we undertook to identify a statin molecule which retained the ability to potently inhibit HMG‐CoA reductase while having no effect on RyR1 ion‐channel function. Hence, a variety of atorvastatin analogues were investigated for their ability to activate RyR1. These were kindly provided by Pfizer (East 42nd Street, New York, 10017, USA) and were based upon Pfizer's previously reported atorvastatin analogue ‘inhibitor 2’ (**P1**, Figure 5a), which differs from atorvastatin through transposition to the nitrogen atom of the pyrrole, and the addition of a second fluoro group as shown in Figure 6a (Pfefferkorn, Song, et al., 2007). The analogues were selected for their structural diversity, and all feature modifications to the hydrophobic region of the atorvastatin molecule, with either bulky or polar groups. We define the hydrophobic region to include both the amide and fluorophenyl substituents of atorvastatin. The chemical structures are shown in Figure 6a. These can be broadly categorised into four series based on their structures: (1) anilide series (**P1** to **P10**), (2) conformational restriction (**P11**), (3) pyridine substituted (**P12** to **P13**) and (3) sulfamoyl pyrroles (**P14** to **P16**).

The synthesis of these analogues has been previously reported and the experimentally determined HMG‐CoA IC_50_ value is available for each molecule. All those included in this study had an HMG‐CoA IC_50_ value lower than 15 nM and hence have proven to be potent statin molecules (Pfefferkorn, Song, et al., 2007). The majority have additionally had this activity confirmed in vivo via a reduction in mouse cholesterol synthesis (Bratton et al., 2007; Larsen et al., 2007; Park et al., 2008; Pfefferkorn, Choi, et al., 2007; Pfefferkorn, Song, et al., 2007; Suzuki et al., 2001).

The analogues were subsequently investigated for their experimental ability to influence RyR1 activity, by examining their ability to increase [^3^H]ryanodine binding to skeletal HSR. As shown in Figure 6b, of the 16 compounds investigated, at the concentration of 100 nM, seven increased (indicating RyR1 activation) and five inhibited (indicating RyR1 inhibition) [^3^H]ryanodine binding. The figure also shows how the ability of atorvastatin and cerivastatin to increase or inhibit [^3^H]ryanodine binding at 100 nM compares with that of the 16 compounds. For the compounds that increased or inhibited [^3^H]ryanodine binding, we investigated the effects of two higher concentrations and the effects of these are shown in Figure 6c,d. Four compounds did not influence [^3^H]ryanodine binding even at concentrations as high as 10 μM as shown in Figure 6d. The effects of cerivastatin (from Figure 1b) are shown to highlight the lack of effect of these compounds.

## 4 DISCUSSION

Despite the widespread use of statin drugs, a clear explanation for their muscle‐related side effects has not yet been established (Ward et al., 2019). We have previously reported that simvastatin significantly and reversibly increased RyR1 Po and suggested that this activation may contribute to the adverse effects of statins, given the crucial role of RyR channels in maintaining cellular Ca^2+^ homeostasis and the pleiotropic consequences of disturbed Ca^2+^ signalling for muscle function (Venturi et al., 2018). In this study, we now find that all statins routinely prescribed in the United Kingdom are RyR1 activators, being capable of significantly increasing RyR1 activity in the concentration range 100–250 nM in the simple [^3^H]ryanodine binding assay and that this can be considered a class effect of statins. Two statins, atorvastatin and cerivastatin, were selected for more detailed investigation, representing statins with low and high prevalence of side effects, respectively (Newman et al., 2019). Cerivastatin was removed from the market due to rhabdomyolysis that resulted in 52 deaths (Furberg & Pitt, 2001) and therefore provides an excellent vehicle to understand the effects of statins on RyR1 channels.

We found that both atorvastatin and cerivastatin increased the Po of RyR1 channels incorporated into planar lipid bilayers, and that this effect was concentration dependent and reversible, as previously observed for simvastatin (Venturi et al., 2018). For atorvastatin, this effect also appeared to be independent of the species from which the RyR1 channels were derived, with mouse, rabbit and sheep RyR1 displaying similar levels of activation (Figure 2d). Lifetime analysis of single‐channel events revealed that, in the presence of activating levels of cytosolic Ca^2+^, atorvastatin also shares the mechanism of activation previously identified for simvastatin. That is, it binds primarily to the closed state of the channel, raising RyR1 Po by increasing the frequency of channel openings. This mechanism is also how cytosolic Ca^2+^ regulates RyR channels (Ashley & Williams, 1990; Sitsapesan & Williams, 1995; Smith et al., 1986). Thus the mechanism for the increase in Po with the binding of low concentrations of simvastatin or atorvastatin to RyR1 may be an increase in the sensitivity of the channel to cytosolic [Ca^2+^] since they are absolutely dependent on the presence of activating levels of cytosolic Ca^2+^ (reducing free cytosolic [Ca^2+^] to <1 nM abolishes low‐concentration simvastatin and atorvastatin‐induced channel openings). In the case of atorvastatin, some Ca^2+^‐independent openings could be induced by high concentrations (100 μM) of statin but only very low Po values could be achieved. On the contrary, even low concentrations of cerivastatin (10 nM) were able to open the channels at sub‐activating cytosolic [Ca^2+^]. Importantly, 10 nM cerivastatin could also significantly activate RyR1 at physiological resting free [Ca^2+^] (100 nM) in the presence of the key physiological RyR1 regulators, Mg^2+^ and ATP. Excess RyR1 Ca^2+^‐leak at rest is a well‐established phenomenon associated with disease states such as central core disease and malignant hyperthermia (Kushnir et al., 2020). Thus, these powerful agonistic effects of cerivastatin on RyR1 are especially noteworthy, given that cerivastatin was withdrawn from the market. Also, cerivastatin, compared to other statins, most effectively increased the binding of [^3^H]ryanodine to isolated SR vesicles, in line with having the greatest ability to increase the Po of individual RyR1 channels. If, as we suggest, RyR1 dysfunction initiates the intracellular changes that lead to statin‐induced myopathy, then we would indeed expect the side‐effects of cerivastatin to be the most severe of the clinically relevant statins. The exact correlation between RyR1 activation and propensity for muscular side effects will also depend on other additional factors, such as the lipophilicity of the statin drug in question, as more lipid‐soluble statins such as cerivastatin have also been shown to penetrate the muscle to a greater extent (Fong, 2014; Schachter, 2005). However, the finding that cerivastatin is the strongest activator of RyR, at all cytosolic [Ca^2+^], including sub‐activating levels, supports the hypothesis that inappropriate activation of RyR1 may be the first step leading to statin‐induced myopathy.

The single‐channel methods employed this study allow the exact concentration of statin in the vicinity of RyR1 to be tightly controlled, an advantage over cellular experiments that rely on a drug to cross cellular membranes. However, in order to fully understand the effects of statins on skeletal muscle, we must determine what concentration of statin the RyR channels may be exposed to in a clinical setting. There is no doubt that the plasma concentrations of statins are low, and for nearly all statins, high plasma protein affinity reduces the ‘free’ concentration further (Stern et al., 2000). In the case of atorvastatin, steady state plasma concentrations have been found to be approximately 4 nM and 10 nM for 20 mg and 80 mg dosing regimens respectively (DeGorter et al., 2013). However, RyR1 is expressed in muscle tissue and it has been found that statins accumulate in the muscles of human patients. Biopsies have shown that atorvastatin and simvastatin are present in muscle tissue at 300 and 490 times their plasma concentration respectively (Schirris et al., 2015). This is also consistent with the fact that their IC_50_ values for HMG‐CoA reductase range from approximately 5 nM to 44 nM (McKenney, 2003), many times their plasma concentrations. This effect is predominately driven by active transporters such as the organic anion transporter OATP2B1 (Knauer et al., 2010; Schirris et al., 2015) and the constant interconversion between their open/acid and more lipophilic lactone forms, allowing the latter to accumulate. For atorvastatin, the concentration reaches a maximum of approximately 120 nM. The fact that we found that atorvastatin and cerivastatin both cause significant RyR1 activation in this range further supports the likelihood that increased RyR1 activation in statin users may be clinically relevant. For atorvastatin these effects are at the higher range of concentrations and therefore may only be relevant for patients prescribed higher doses of statins, such as following myocardial infarction. However, the finding that cerivastatin significantly activated RyR1 at even low nanomolar concentration would seem to be consistent with the more prevalent side effects seen with this withdrawn statin (Furberg & Pitt, 2001).

Having found that all commonly prescribed statins are agonists of RyR1, we investigated if this was an activity shared by all competitive inhibitors of HMG‐CoA reductase. If undesired RyR1 activation gives rise to statin myopathy, the design of new statin drugs with reduced side effects would require that the functional groups of the molecule which cause RyR1 activation can be removed without disturbing its affinity for HMG‐CoA reductase. All competitive inhibitors of HMG‐CoA reductase require the minimum essential statin moiety, (*R*)‐**1**, described in Figure 5a; thus, the design of new statin drugs requires that this motif is retained. We therefore investigated if this motif itself contained the ability to activate RyR1. Other small negatively charged molecules such as inorganic phosphate (Pi) and triphosphate (PPPi) (Fruen et al., 1994; Lindsay et al., 2018) have previously been shown to activate RyR channels. We found that neither the open or closed ring forms of (*R*)‐**1** (Figure 5a) had any effect on RyR1 activity at concentrations up to 1 mM. This cannot, of course, be a direct comparison as the isolated (*R*)‐**1** unit has a much lower molecular weight than the statin molecules it mimics, and has fewer functional groups to create positive molecular interactions. However, the fact that (*R*)‐**1** was inactive even at millimolar concentrations suggests that this essential statin unit is not, on its own, the motif responsible for binding to RyR1 or activating the channel. This suggested that it may be possible for a molecule to be a potent inhibitor of HMG‐CoA reductase while lacking RyR1 activity.

This finding is also consistent with the observation that myotoxicity appears to be associated with the inactive lactone form of statins, which would not be the case if it was a direct result of HMG‐CoA inhibition. One can therefore speculate that the increased myotoxicity observed with the lactone form may be a result of its enhanced cellular update into skeletal muscle cells where RyR1 is heavily expressed (Taha et al., 2016).

We extended this finding by screening a structurally diverse collection of atorvastatin analogues for RyR1 activity, and identified four analogues that were potent inhibitors of HMG‐CoA reductase (IC_50_ < 2 nM) and which did not activate or inhibit RyR1 at concentrations which are many times greater than that required to provide the desired inhibition of HMG‐CoA reductase. Concentrations as high as 1 mM did not influence RyR1 activity and this is approximately 10^6^ times greater than the HMG‐CoA IC_50_ for compounds **P1**, **P5**, **P6** and **P8** (1.8 nM, 0.2 nM, 0.3 nM and 0.8 nM, respectively) (Pfefferkorn, Song, et al., 2007). For comparison, atorvastatin and cerivastatin already activate RyR1 to an extent at concentrations equal to their HMG‐CoA IC_50_ (8–10 nM) (Figure 2). Importantly, these analogues have already been shown to possess in vivo cholesterol‐lowering effects, as well as an acceptable safety profile (Bratton et al., 2007; Larsen et al., 2007; Park et al., 2008; Pfefferkorn, Choi, et al., 2007; Pfefferkorn, Song, et al., 2007; Suzuki et al., 2001). These compounds therefore demonstrate that RyR1 activation is not a property dooming all statins but is a separate and surmountable obstacle.

**FIGURE 7:**
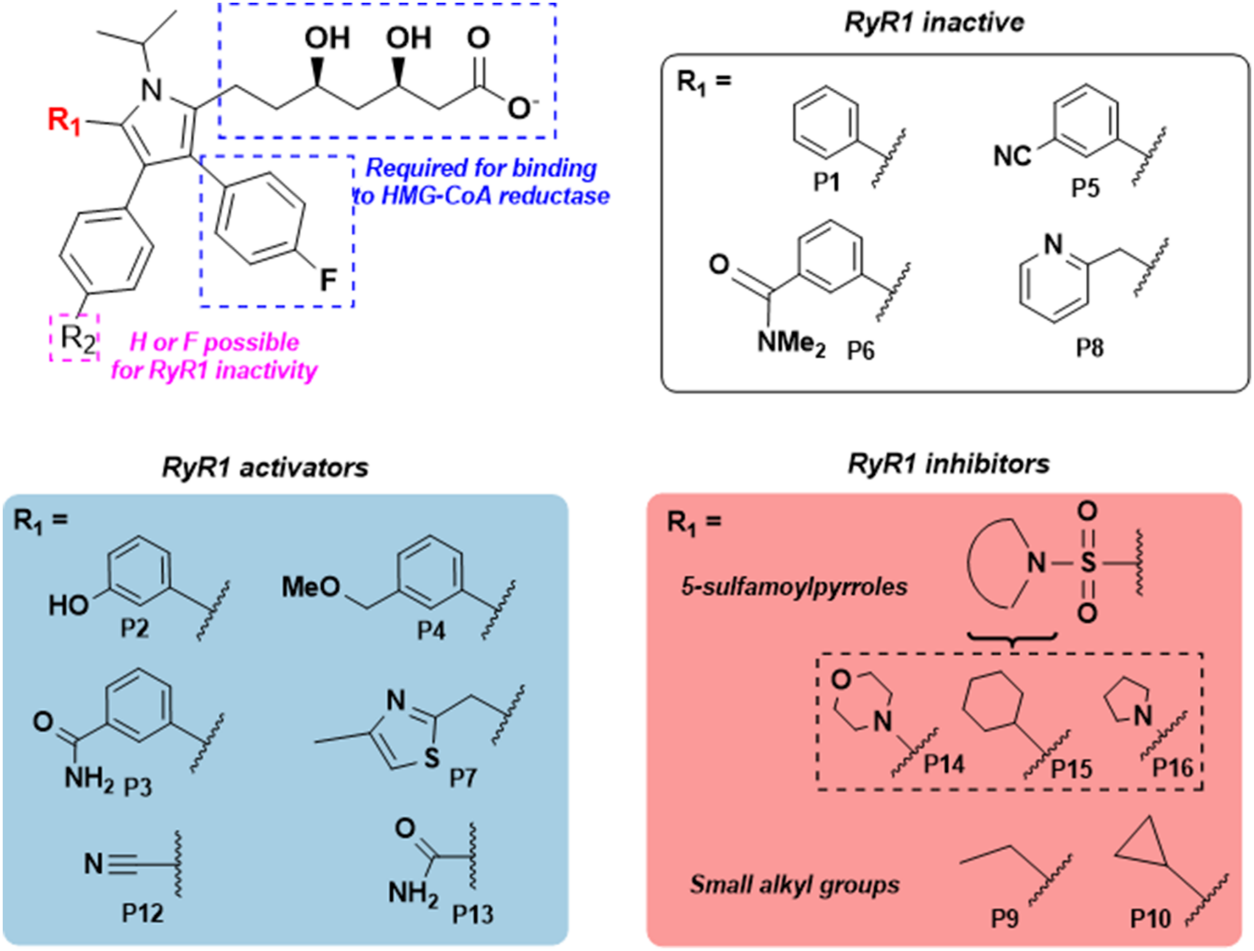
General structure–activity relationships showing the structural features which influenced ryanodine RyR1 activity. Panels show how the identity of the R_1_ group corresponds with the ability to activate or inhibit RyR1 or lacks ability to affect RyR1 Po as indicated. Compound numbers are also indicated below each structure

Our results have indicated several structural features of statin molecules that may drive activation and inhibition of RyR1. These features are summarised in Figure 7. Principally, it appears that compounds possessing a small polar functionality at C(5) of the pyrrole (**P12**, cyano and **P13**, aminocarbonyl) appeared to be the activators of RyR1, including an example where the amide was cyclised onto the adjacent aryl substituent at C(4). Conversely, the three compounds that possess a 5‐sulfamoyl pyrrole motif (**P14**, **P15** and **P16**) were uniformly inhibitors, with all three reducing [^3^H]ryanodine binding to around 50% of control levels. Analogues with small groups at C(5) of the pyrrole such as ethyl or cyclopropyl groups (**P9** and **P10**) were also inhibitors. It is also conspicuous that cerivastatin is alone in being the only statin based upon a pyridine core. Contrasting this with similar pyrimidine core of rosuvastatin leads us to speculate that the pyridine core is instrumental in causing cerivastatin to be such an effective RyR1 activator.

However, the most important finding of this work has been the identification of compounds which are inactive against RyR1, and, in this regard, it is conspicuous that all of the inactive compounds (**P1**, **P5**, **P6** and **P8**) have in common a C(5) aryl ring, although further structure–activity‐relationship analyses will be required to understand which substituents and regiochemistry are critical to avoid RyR1 activation. This suggests that it may be this functionality that is important for reducing a compound's ability to bind to or activate the channel, and other groups such as the 4‐sulfamoyl pyrrole can bind more easily. Importantly, these inactive compounds retain the functionality required for binding to HMG‐CoA reductase, including the essential (*R*)‐**1** unit and the 4‐fluorophenyl group. While further studies will be required to understand the cause of this structure–activity relationship, these trends represent important new information for the development of new RyR1 inactive statin compounds.

In summary, we have demonstrated that all clinically used statins in the United Kingdom are potent partial agonists of RyR1, capable of increasing the Po of RyR1. This could be an initiating factor in statin‐induced myopathy. We show that cerivastatin additionally possesses a unique ability to activate RyR1 in the absence of activating levels of cytosolic [Ca^2+^], making the channel more likely to leak excess SR Ca^2+^ at rest, causing patients to be at increased risk of rhabdomyolysis and death. We have also identified potent inhibitors of HMG‐CoA reductase that can inhibit RyR1 or, importantly, do not modulate RyR1 Po at all. This crucial finding, that statin‐modulation of RyR1 and HMG‐CoA are clearly separable effects, will facilitate the development of novel statin compounds that do not target RyR1, thus reducing the likelihood of dysfunctional muscle Ca^2+^‐handling. With increasing numbers of patients becoming eligible for statin therapy, it is more important than ever to facilitate the development of next‐generation statins that will allow more patients to benefit from their cardioprotective effects.

## CONFLICT OF INTEREST

The authors declare no competing financial interests.

## AUTHOR CONTRIBUTIONS

C.L. performed the experiments and analysed the data. All authors conceived and designed the study, wrote the manuscript, discussed the results and critiqued the manuscript for intellectual content.

## DECLARATION OF TRANSPARENCY AND SCIENTIFIC RIGOUR

This Declaration acknowledges that this paper adheres to the principles for transparent reporting and scientific rigour of preclinical research as stated in the BJP guidelines for Design and Analysis, and Animal Experimentation, and as recommended by funding agencies, publishers and other organisations engaged with supporting research.

## Supporting information


**Figure S1.** **Synthetic route to statin structural units (R)‐1 and (S)‐1**
Reaction conditions: (i) NaH, TBDPSCl, THF, RT, 16 h; (ii) (COCl)_2_, DMSO, Et_3_N, ‐78 °C, 1 hr; (iii) **4**, nBu_2_BOTf, CH_2_Cl_2_, ‐78 °C to 0 °C, 2.5 h; (iv) HF/pyr, THF, RT, 3 h; (v) Et_3_N, CH_2_Cl_2_, RT, 48 h
**Table S1. The effects of atorvastatin on channel lifetime parameters** Time constants (T1, T2, T3, T4) and percentage areas (A1, A2, A3, A4) obtained from maximum likelihood fitting of pdfs to open and close lifetime distributions of 5 independent single RyR1 channels in the presence and absence of 10 μM atorvastatin are shown.

## Data Availability

The data that support the findings of this study are available from the corresponding author upon reasonable request.
