# Supplementary material for: Statin activation of skeletal ryanodine receptors (RyR1) is a class effect but separable from HMG‐CoA reductase inhibition
Source: Br J Pharmacol. 2022 Aug 2;179(21):4941–57. doi: 10.1111/bph.15893 (PMC9804224; doi:10.1111/bph.15893)
Supplement: Supplementary file 1 — Figure S1. Synthetic route to statin structural units (R)‐1 and (S)‐1 Reaction conditions: (i) NaH, TBDPSCl, THF, RT, 16 h; (ii) (COCl)2, DMSO, Et3N, ‐78 °C, 1 hr; (iii) 4, nBu2BOTf, CH2Cl2, ‐78 °C to 0 °C, 2.5 h; (iv) HF/pyr, THF, RT, 3 h; (v) Et3N, CH2Cl2, RT, 48 h Table S1. The effects of atorvastatin on channel lifetime parameters Time constants (T1, T2, T3, T4) and percentage areas (A1, A2, A3, A4) obtained from maximum likelihood fitting of pdfs to open and close lifetime distributions of 5 independent single RyR1 channels in the presence and absence of 10 μM atorvastatin are shown. [file BPH-179-4941-s001.docx]

**Statin-activation of RyR1 is a class effect but separable from HMG-CoA reductase inhibition**

Chris Lindsay^1^, Maria Musgaard^3,4^, Angela J. Russell^1,2^ & Rebecca Sitsapesan^1*^

^1^ Department of Pharmacology, University of Oxford, UK

^2^ Department of Chemistry, Chemistry Research Laboratory, University of Oxford, UK

^3^ Structural Bioinformatics and Computational Biochemistry, Department of Biochemistry, University of Oxford, UK.

^4^ Present address: OMass Therapeutics, The Schrödinger Building, Heatley Road, The Oxford Science Park, Oxford, OX4 4GE, UK

**Supplementary Figures**

**Figure S1. Synthetic route to statin structural units (R)-1 and (S)-1**

Reaction conditions: (i) NaH, TBDPSCl, THF, RT, 16 h; (ii) (COCl)_2_, DMSO, Et_3_N, -78^o^C, 1hr; (iii) **4**, nBu_2_BOTf, CH_2_Cl_2_, -78^o^C to 0^o^C, 2.5 h; (iv) HF/pyr, THF, RT, 3 h; (v) Et_3_N, CH_2_Cl_2_, RT, 48 h

**Supplementary Tables**

**Table S1. The effects of atorvastatin on lifetime parameters**

**Open Times**

|  | **10 μM Ca^2+^** | | | | | | | | **after 10 μM atorvastatin** | | | | | | | |
| --- | --- | --- | --- | --- | --- | --- | --- | --- | --- | --- | --- | --- | --- | --- | --- | --- |
|  | T1 (ms) | A1 (%) | T2  (ms) | A2 (%) | T3 (ms) | A3  (%) | T4 (ms) | A4  (%) | T1 (ms) | A1  (%) | T2  (ms) | A2  (%) | T3 (ms) | A3  (%) | T4  (ms) | A4  (%) |
| **channel 1** | 1.0 | 100 |  |  |  |  |  |  | 1.0 | 93 | 6.0 | 7 |  |  |  |  |
| **channel 2** | 1.0 | 94 | 1.5 | 6 |  |  |  |  | 1.0 | 100 |  |  |  |  |  |  |
| **channel 3** | 1.0 | 100 |  |  |  |  |  |  | 1.0 | 87 | 8.5 | 13 |  |  |  |  |
| **channel 4** | 1.0 | 100 |  |  |  |  |  |  | 1.0 | 92 | 9.9 | 8 |  |  |  |  |
| **channel 5** | 1.0 | 100 |  |  |  |  |  |  | 1.4 | 81 | 9.0 | 19 |  |  |  |  |
|  | **Closed Times** | | | | | | | | | | | | | | | |
|  | **10 μM Ca^2+^** | | | | | | | | **after 10 μM atorvastatin** | | | | | | | |
|  | T1 (ms) | A1 (%) | T2  (ms) | A2 (%) | T3 (ms) | A3  (%) | T4 (ms) | A4  (%) | T1 (ms) | A1  (%) | T2  (ms) | A2  (%) | T3 (ms) | A3  (%) | T4  (ms) | A4  (%) |
| **channel 1** | 2.2 | 75 | 16.6 | 23 | 761.4 | 2 |  |  | 1.0 | 89 | 7.8 | 10 | 227.3 | 1 |  |  |
| **channel 2** | 4.5 | 41 | 34.6 | 58 | 661.0 | 1 |  |  | 2.1 | 83 | 14.9 | 17 |  |  |  |  |
| **channel 3** | 4.2 | 33 | 36.4 | 60 | 190.5 | 7 |  |  | 1.0 | 76 | 7.8 | 23 | 218.6 | 1 |  |  |
| **channel 4** | 1.0 | 34 | 5.5 | 32 | 31.0 | 27 | 81.1 | 6 | 1.0 | 77 | 2.5 | 20 | 18.1 | 3 |  |  |
| **channel 5** | 2.9 | 43 | 21.7 | 49 | 173.1 | 8 |  |  | 1.0 | 80 | 1.8 | 20 |  |  |  |  |

**Table S1. The effects of atorvastatin on lifetime parameters** Time constants (T1, T2, T3, T4) and percentage areas (A1, A2, A3, A4) obtained from maximum likelihood fitting of pdfs to open and close lifetime distributions of 5 independent single RyR1 channels in the presence and absence of 10 µM atorvastatin are shown.

**Supplementary Methods**

**General Experimental Methods**

All reactions involving moisture-sensitive reagents were performed using oven dried glassware under an atmosphere of nitrogen using anhydrous solvents. Anhydrous solvents were dried using a protocol modified from that previously reported (Pangborn et al., 1996), all other reagents were used without further purification unless otherwise stated. ‘Petrol’ refers to the 30-40^o^C fraction of petroleum ether. Thin layer chromatography was performed using Merck silica gel 60 F_254_ thin layer chromatography sheets, supported by aluminium backs. Plates were visualised either by UV light (254 nM) or thermal development following exposure to 1% KMnO_4_ or *p*-anisaldehyde. Flash column chromatography was performed using Kieselgel 60 silica in a glass column. Optical rotations were recorded on a Perkin-Elmer 241 polarimeter with a water-jacked 10 cm cell at the temperature indicated. Specific rotations are reported in 10^-1^ deg cm^2^ g^-1^ and concentrations in g/100 ml. Infrared spectra were recorded on a Bruker Tensor 27 FT-IR spectrometer using thin film samples dissolved in CHCl_3_. Selected characteristic peaks above 1500 cm^-1^ are reported in wavenumbers (cm^-1^). High resolution mass spectra (HRMS) were recorded on a Bruker MicroTOF. ^1^H NMR spectra were recorded on a Bruker AV400 (400 MHz) spectrometer and referenced to residual solvent peaks. Chemical shifts are quoted in parts per million (ppm). Coupling constants J (Hz) are recorded to the nearest 0.5 Hz. Where peak multiplicities are reported, the following abbreviations are used: s = singlet, d = doublet, t = triplet, q = quartet, m = multiplet, br = broadened, dd = doublet of doublets, ddd= double doublet of doublets.

**3-((*tert*-butyldiphenylsilyl)oxy)propan-1-ol**, **(3)**

NaH (570 mg, 13.2 mmol, 1.0 eq) was suspended in THF (60 mL) under nitrogen and cooled to 0^o^C. A solution of 1,3-propanediol (0.950 mL, 13.2 mmol, 1.0 eq) in THF (10 ml) was added dropwise. The reaction mixture was warmed to room temperature and allowed to stir for 16 h. The reaction mixture was again cooled to 0^o^C before the dropwise addition of *tert*-butyl(chloro)diphenylsilane (3.70 mL, 13.2 mmol, 1 eq). After stirring for a further 2 h at 0^o^C, the reaction was quenched by adding crushed ice (100 g), allowed to warm to room temperature and diluted with EtOAc (25 mL). The layers were separated, and the aqueous layer extracted with EtOAc (25 mL). The combined organic layers were washed with water, brine, dried (MgSO_4_) and concentrated under reduced pressure. Purification by flash column chromatography (eluent EtOAc/petrol, 25:75) gave **3** as a white solid (3.49 g, 84%); **mp** 39-42^o^C (lit 42-43^o^C, EtOAc) (Zurwerra et al., 2012); TLC (SiO_2_): **R_f_** = 0.40 (3:7 ethyl acetate:cyclohexane); **^1^H NMR** (400 MHz, CDCl_3_): *δ* 7.71-7.66 (m, 5H, Ph*H*), 7.48-7.36 (m, 5H, Ph*H*), 3.85 (td, *J* = 5.7, 1.6 Hz, 4H, *H_2_*, *H_3_*), 2.38 (s, 1H, O-*H*), 1.82 (q, *J* = 5.7 Hz, 2H, *H_2_*), 1.06 (s, 9H, C(C*H_3_*)_3_); **^13^C NMR** (101 MHz, CDCl_3_): *δ* 135.4 (Ph), 133.1 (Ph), 129.6 (*C*_2_), 61.8 (*C*_3_), 34.1 (*C*_4_), 26.7 (*C*(CH_3_)_3_), 18.9 (C(*C*H_3_)_3_); ***m/z* (ESI^+^)** 337; **HRMS**: C_19_H_26_O_2_NaSi requires 337.15943; found 337.15948. Spectral data were found to be consistent with literature values (Raghavan and Samanta, 2013).

**3-((tert-butyldiphenylsilyl)oxy)propanal**, **(4)**

Oxalyl chloride (1.20 mL, 14.3 mmol, 1.5 eq) was added to CH_2_Cl_2_ (60 mL) under nitrogen and the solution was cooled to -78^o^C. DMSO (2.20 mL, 30.6 mmol, 3.2 eq) was added slowly dropwise and the solution was allowed to stir for 5 min_._ A solution of compound **3** (3.00 g, 9.54 mmol, 1.0 eq) in CH_2_Cl_2_ (20 mL) was added dropwise. The reaction mixture was stirred for 15 min at -78^o^C before addition of triethylamine (6.9 mL, 47.7 mmol, 5.0 eq). The reaction mixture was allowed to warm to room temperature, water (50 mL) was added and layers were separated. The aqueous layer was extracted with CH_2_Cl_2_ (3 x 15 mL). The combined organic layers were washed with 1 M HCl (aq., 2 x 50 mL), brine and dried (MgSO_4_). The solvent was removed under reduced pressure. Purification by flash column chromatography (eluent EtOAc/petrol, 10:90) gave **4** as a pale yellow oil, which solidified after drying under high-vacuum (2.69 g, 90%). TLC (SiO_2_): **R_f_** = 0.60 (3:7 EtOAc:cyclohexane); **^1^H NMR** (400 MHz, CDCl_3_): *δ* 9.83 (t, *J* = 2.2 Hz, 1H, *H_2_*), 7.71-7.63 (m, 5H, Ph*H*), 7.50-7.36 (m, 5H, Ph*H*), 4.03 (t, *J* = 6.0 Hz, 2H, *H_3_*), 2.61 (td, *J* = 6.0, 2.2 Hz, 2H, *H_4_*), 1.05 (s, 9H, C(C*H_3_*)_3_); **^13^C NMR** (101 MHz, CDCl_3_): *δ* 201.7 (*C*_2_), 135.4 (Ph), 133.1 (Ph), 58.1 (*C*_3_), 46.2 (*C*_4_), 26.6 (*C*(*C*H_3_)_3_), 19.0 (C(*C*H_3_)_3_); ***m/z* (ESI^+^)** 335; **HRMS**: C_19_H_24_O_2_Na^28^Si requires 335.14378; found 335.14384. Spectral data were found to be consistent with literature values (Raghavan and Samanta, 2013).

**(*R*)-4-benzoyl-3-(*R/S*)-5-((*tert*-butyldiphenylsilyl)oxy)-3-hydroxypentanoyloxazolidin-2-one (6) and (7)**

Acetyl-4-benzyl oxazolidine (1.00 g, 4.56 mmol, 1.0 eq) was added to a flame-dried flask under nitrogen and dissolved in dichloromethane (degassed (x3), 20 mL) before cooling to −78^o^C. The resulting solution was treated with dibutylboron triflate (1.0 M in dichloromethane, 0.83 mL, 6.02 mmol, 1.32 eq), followed by dropwise addition of triethylamine (787 µL, 5.70 mmol, 1.32 eq.) at such a rate as to keep the internal temperature below 3°C. The solution turned slightly yellow or green during the dibutylboron triflate addition, and then to light yellow when triethylamine is added. When the internal temperature dropped below −65°C, a solution of aldehyde **4 (**1.56 g, 5.11 mmol, 1.11 eq) in dichloromethane (20 mL) was added. The resulting solution was stirred for 5 h at −78^o^C, then quenched by addition of a pH 7.4 aqueous phosphate saline buffer (5 mL) and methanol (14 mL) and allowed to warm to 0^o^C. A solution of methanol and 30% aqueous hydrogen (2:1, 14 mL) was then added at such a rate as to keep the internal temperature below 10°C. The resulting mixture was stirred for a further 1 h, the solvent was partially removed on a rotary evaporator at a bath temperature of 25–30°C. The resulting slurry was extracted diethyl ether (3 x 25 mL). The combined organic extracts were washed with aq. saturated sodium bicarbonate, brine and dried (MgSO_4_). The solvent was removed under reduced pressure. Purification by flash column chromatography (eluent EtOAc/40-60^o^C petrol, 10:90 🡪 25:75) gave **6** (1.57 g, 38%) and **7** (925 mg, 23%) as colourless oils.

TLC (SiO_2_): **R_f_** = 0.65 (3:7 EtOAc:cyclohexane) **IR** ν_max_ (film): 3512 (O-H), 1781 (C=O), 1696 (C=O), 1103 (C-N); **^1^H NMR** (400 MHz, CDCl_3_) *δ* 7.75 – 7.63 (m, 4H, Ph*H*), 7.50 – 7.42 (m, 3H, Ph*H*), 7.41 – 7.39 (m, 2H, Ph*H*), 7.38 – 7.31 (m, 2H, Ph*H*), 7.33 – 7.25 (m, 2H, Ph*H*), 7.24 – 7.18 (m, 2H, Ph*H*), 4.76 – 4.64 (m, 1H, *H_4_*), 4.45 (m, *J* = 4.1 Hz, 1H, *H_11_*), 4.26 – 4.07 (m, 2H, *H_3_*), 3.97 – 3.82 (m, 2H, *H_10_*), 3.45 (s, 1H, O-*H*), 3.30 (dd, *J* = 13.5, 3.3 Hz, 1H, *H_7_*), 3.21 – 3.12 (m, 2H, *H_13_*), 2.79 (dd, *J* = 13.4, 9.5 Hz, 1H, *H_7_*), 1.91 – 1.71 (m, 2H, *H_12_*), 1.06 (s, 9H, 3 x C*H_3_*);**^13^C NMR** (101 MHz, CDCl_3_): *δ* 201.7 (*C*_8_), 135.4 (*C*_1_), 133.1 (Ph), 129.6 (Ph), 127.6 (Ph), 127.6 (Ph), 58.1 (*C*_3_), 52.1 (*C*_11_) 46.2 (*C*_13_), 41.4 (*C*_4_), 41.2 (*C*_10_), 36.6 (*C*_7_) 19.0 (*C*_12_); ***m/z* (ESI^+^)** 532; **HRMS:** C_31_H_38_O_5_NSi requires 532.25138; found 532.25179.

TLC (SiO_2_): **R_f_** = 0.60 (3:7 EtOAc:cyclohexane)*;* **IR** ν_max_ (film): 3034 (O-H), 1826 (C=O), 1781 (C=O), 1104 (C-N); **^1^H NMR** (400 MHz, CDCl_3_): δ 7.73 – 7.63 (m, 4H, Ph*H*), 7.48 – 7.41 (m, 3H, Ph*H*), 7.41 – 7.37 (m, 2H, Ph*H*), 7.37 – 7.31 (m, 2H, Ph*H*), 7.31 – 7.25 (m, 2H, Ph*H*), 7.25 – 7.18 (m, 2H, Ph*H*), 4.76 – 4.65 (m, 1H, *H_4_*), 4.48 (p, *J* = 4.2 Hz, 1H, *H_11_*), 4.26 – 4.07 (m, 2H, *H_3_*), 3.97 – 3.82 (m, 2H, *H_10_*), 3.45 (s, 1H, O-*H*), 3.32 (dd, *J* = 13.4, 3.4 Hz, 1H, *H_7_*), 3.21 – 3.12 (m, 2H, *H_13_*), 2.79 (dd, *J* = 13.4, 9.6 Hz, 1H, *H_7_*), 1.91 – 1.71 (m, 2H, *H_12_*), 1.06 (s, 9H, 3 x C*H*_3_); **^13^C NMR** (101 MHz, CDCl_3_): *δ* 201.7 (*C*_8_), 135.4 (*C*_1_), 133.1 (Ph), 129.6 (Ph), 127.6 (Ph), 127.6 (Ph), 58.1 (*C*_3_), 46.2 (*C*_13_), 41.3 (*C*_4_), 41.2 (*C*_10_), 36.6 (*C*_7_), 19.0 (*C*_12_)***; m/z* (ESI^+^)** 532; **HRMS:** C_31_H_38_O_5_NSi requires 532.25138; found 532.25269. Spectral data were found to be consistent with literature values (Loubinoux et al., 1995)

**(*R*)-4-benzoyl-3-((*R*)-3,5-dihydroxypentanoyl)oxazolidin-2-one**, **(8)**

HF/pyr (118 µL, 4.13 mmol, 5.0 eq) was added slowly to an ice-cold solution of imide **6** (440 mg, 0.827 mmol, 1.0 eq) in THF (15 mL) in a falcon tube. The resulting mixture was stirred at 0^o^C for 4 h. The falcon tube was opened to air, and aq. saturated sodium bicarbonate was added dropwise until no further gas could been seen evolving. The organic layer was extracted with EtOAc (2 x 20 mL), and washed with HCl (aq., 1M), NaHCO_3_ (aq. sat. sol.) and brine. The organic layer was dried (MgSO_4_) and concentrated under reduced pressure. Purification by flash column chromatography (eluent 75% 🡪 100% EtOAc; cyclohexane) gave alcohol **8** as a colourless oil (165 mg, 65%). TLC (SiO_2_): **R_f_** = 0.70 (100% EtOAc); **IR** ν_max_ (film): 3396 (O-H), 2929 (O-H), 1774 (C=O), 1694 (C=O); **^1^H NMR** (400 MHz, CDCl_3_) *δ* 7.43 – 7.31 (m, 2H, Ph*H*), 7.31 – 7.24 (m, 2H, Ph*H*), 7.24 – 7.16 (m, 1H, Ph*H*), 4.71 (ddt, *J* = 9.3, 7.0, 3.3 Hz, 1H, *H_4_*), 4.39 (dddd, *J* = 9.1, 8.0, 4.6, 3.6 Hz, 1H, *H_3_*), 4.28 – 4.20 (m, 1H, *H_11_*), 3.88 (td, *J* = 4.9, 4.4, 1.3 Hz, 2H, *H_7_*), 3.29 (dd, *J* = 13.5, 3.5 Hz, 1H, *H_3_*), 3.21 – 3.06 (m, 2H, *H_12_*), 2.81 (dd, *J* = 13.5, 9.4 Hz, 2H, *H_7_*), 1.92 – 1.71 (m, 2H, *H_10_*), OHs were not observed; **^13^C NMR** (101 MHz, CDCl_3_): *δ* 172.8 (*C*_8_), 135.1 (*C*_1_), 129.6 (Ph), 129.2 (Ph), 127.6 (Ph), 77.5 (Ph), 77.2 (*C*_3_), 76.8 (*C*_11_), 68.1 (*C*_13_), 66.6 (*C*_4_), 61.3 (*C*_10_), 55.2 (*C*_7_), 42.9 (*C*_12_); ***m/z* (ESI^+^)** 316; **HRMS:** C_15_H_19_O_5_NNa requires 316.11554; found 316.11535; Spectral data were found to be consistent with literature values (Loubinoux et al., 1995)

**(*R*)-4-benzoyl-3-((*S*)-3,5-dihydroxypentanoyl)oxazolidin-2-one**, **(9)**

Using a similar procedure to the synthesis of alcohol **8**, from imide **6** (440 mg, 0.827 mmol) **9** was obtained as a colourless oil (147 mg, 59%). TLC (SiO_2_): **R_f_** = 0.70 (100% EtOAc); **IR** ν_max_ (film): 3396 (O-H), 2929 (O-H), 1774 (C=O), 1694 (C=O). **^1^H NMR** (400 MHz, CDCl_3_) *δ* 7.45 – 7.31 (m, 2H, Ph*H*), 7.38 – 7.24 (m, 2H, Ph*H*), 7.23 – 7.15 (m, 1H, Ph*H*), 4.71 (ddt, *J* = 9.2, 7.0, 3.3 Hz, 1H, *H_4_*), 4.40 (m, 1H, *H_3_*), 4.28 – 4.23 (m, 1H, *H_11_*), 3.88 (td, *J* = 4.9, 4.5, 1.3 Hz, 2H, *H_7_*), 3.30 (dd, *J* = 13.5, 3.5 Hz, 1H, *H_3_*), 3.20 – 3.06 (m, 2H, *H_12_*), 2.81 (dd, *J* = 13.5, 9.4 Hz, 2H, *H_7_*), 1.92 – 1.71 (m, 2H, *H_10_*), OHs were not observed; **^13^C NMR** (101 MHz, CDCl_3_): *δ* 172.8 (C_8_), 135.1 (C_1_), 129.6 (Ph), 129.2 (Ph), 127.6 (Ph), 77.5 (Ph), 77.2 (C_3_), 76.8 (C_11_), 68.1 (C_13_), 66.6 (C_4_), 61.3 (C_10_), 55.2 (C_7_), 42.9 (C_12_); ***m/z* (ESI^+^)** 316; **HRMS:** C_15_H_19_O_5_NNa requires 316.11554; found 316.11564**.** Spectral data were found to be consistent with literature values (Loubinoux et al., 1995).

**(*R*)-4-hydroxytetrahydro-2*H*-pyran-2-one, (R)-1**

Triethylamine (0.28 mL, 1.36 mmol, 6.0 eq) was added dropwise to a solution of alcohol **8** (80 mg, 0.272 mmol, 1.0 eq) in CH_2_Cl_2_ (5 mL) and the mixture allowed to stir at room temperature for 16 h. When TLC indicated complete consumption of the starting material (R_f_ = 0.7, 100% EtOAc), toluene (2 mL) was added and the mixture concentrated under reduced pressure. After co-evaporation with 2 further aliquots of toluene, purification by flash column chromatography (eluent 50% 🡪 100% EtOAc: pentane) gave **R-1** as a colourless oil (16 mg, 51%). TLC (SiO_2_): **R_f_** = 0.52 (100% EtOAc); $\boldsymbol{[\alpha]}_{\boldsymbol{D}}^{\boldsymbol{25}}$ = - 0.60 (CHCl_3_, c = 0.1); **IR** ν_max_ (film): 3386 (O-H), 1717 (C=O); **^1^H NMR** (400 MHz, CDCl_3_): *δ* 4.57 (ddd, J = 11.4, 8.2, 4.2 Hz, 1H, *H_4_*), 4.41 – 4.17 (m, 2H, *H_6_*), 2.81 (ddd, J = 17.6, 5.3, 0.9 Hz, 1H, *H_3_*), 2.61 (m, 1H, *H_3_*), 2.11 (ddd, J = 13.7, 8.4, 4.7 Hz, 1H, *H_5_*), 1.89 (dtdd, J = 14.1, 6.4, 4.2, 1.2 Hz, 1H, *H_5_*), OH was not observed; **^13^C NMR** (101 MHz, CDCl_3_): *δ* 170.8 (C_2_=O), 77.8 (C_4_), 77.5 (C_6_), 65.8 (C_5_), 63.7 (C_3_); ***m/z* (ESI^+^)** 117; **HRMS:** C_5_H_9_O_3_ requires 117.05462; found 117.05480. Spectral data were found to be consistent with literature values (Loubinoux et al., 1995).

**(*S*)-4-hydroxytetrahydro-2*H*-pyran-2-one, (S)-1**

Triethylamine (0.28 mL, 1.36 mmol, 6.0 eq) was added dropwise to a solution of alcohol **9** (80 mg, 0.272 mmol, 1.0 eq) in CH_2_Cl_2_ (5 mL) and the mixture allowed to stir at room temperature for 16 h. When TLC indicated complete consumption of the starting material (R_f_ = 0.7, 100% EtOAc), toluene (2 mL) was added, and the mixture concentrated under reduced pressure. After co-evaporation with 2 further aliquots of toluene, purification by flash column chromatography (eluent 50% 🡪 100% EtOAc: pentane) gave **S-1** as a colourless oil (15 mg, 49%). TLC (SiO_2_): **R_f_** = 0.54 (100% EtOAc); $\boldsymbol{[\alpha]}_{\boldsymbol{D}}^{\boldsymbol{25}}$ = + 2.70 (CHCl_3_, c = 0.1); **IR** ν_max_ (film): 3381 (O-H), 1716 (C=O); **^1^H NMR** (400 MHz, CDCl_3_): *δ* 4.57 (ddd, J = 11.3, 8.2, 4.2 Hz, 1H, *H_4_*), 4.42 – 4.16 (m, 2H, *H_6_*), 2.81 (ddd, J = 17.5, 5.3, 0.9 Hz, 1H, *H_3_*), 2.60 (m, 1H, *H_3_*), 2.10 (ddd, J = 13.6, 8.4, 4.7 Hz, 1H, *H_5_*), 1.90 (m, 1H, *H_5_*), OH was not observed; **^13^C NMR** (101 MHz, CDCl_3_) δ 170.8 (C_2_=O), 77.8 (C_4_), 77.5 (C_6_), 65.8 (C_5_), 63.7 (C_3_); ***m/z* (ESI^+^)** 117; **HRMS:** C_5_H_9_O_3_ requires 117.05462; found 117.05473. Spectral data were found to be consistent with literature values (Loubinoux et al., 1995).

**References**

Loubinoux, B., Sinnes, J.L., Osullivan, A.C., and Winkler, T. (1995). The Enantioselective Synthesis of Simplified Southern-Half Fragments of Soraphen-A. Tetrahedron *51*: 3549–3558.

Pangborn, A.B., Giardello, M.A., Grubbs, R.H., Rosen, R.K., and Timmers, F.J. (1996). Safe and convenient procedure for solvent purification. Organometallics *15*: 1518–1520.

Raghavan, S., and Samanta, P.K. (2013). Stereoselective synthesis of the C13-C28 subunit of (-)-laulimalide utilizing an α-chlorosulfide intermediate. Synlett *24*: 1983–1987.

Zurwerra, D., Glaus, F., Betschart, L., Schuster, J., Gertsch, J., Ganci, W., et al. (2012). Total synthesis of (-)-zampanolide and structure-activity relationship studies on (-)-dactylolide derivatives. Chem. - A Eur. J. *18*: 16868–16883.
